# Predicting Mathematics Achievement in Secondary Education: The Role of Cognitive, Motivational, and Emotional Variables

**DOI:** 10.3389/fpsyg.2020.00876

**Published:** 2020-05-26

**Authors:** Amanda Abín, José Carlos Núñez, Celestino Rodríguez, Marisol Cueli, Trinidad García, Pedro Rosário

**Affiliations:** ^1^Department of Psychology, Universidad de Oviedo, Oviedo, Spain; ^2^Facultad de Ciencias Sociales y Humanidades, Universidad Politécnica y Artística de Paraguay, Asunción, Paraguay; ^3^Department of Applied Psychology, Escola de Psicologia, Universidade do Minho, Campus de Gualtar, Braga, Portugal

**Keywords:** intellectual abilities, perceived usefulness, perceived competence, anxiety, intrinsic motivation, achievement motivation, mathematics achievement

## Abstract

Academic achievement in general, and in mathematics in particular, is positively associated not only with cognitive abilities, but also with emotional and motivational skills. The objective of this study was to analyze the prediction strength of cognitive, motivational, and emotional variables in mathematics achievement throughout high school, considering students’ gender and age. A large sample of 2,365 Spanish students from the 4 years of high school (12–16 years old) participated in the study. Students provided information about their intellectual skills, perceived competence in mathematics, perceived utility of mathematics, intrinsic interest in learning, mathematics anxiety, and their causal attributions (for failure and for success), and of their achievement in mathematics. Data showed differences according to gender and the school grade level. The motivational and affective variables did not seem to play an important role in this relationship as predicted in the current study. The results of this study are discussed in light of previous research.

## Introduction

Researchers’ growing interest in studying mathematical achievement is driven by the importance of mathematics in both formal education and people’s daily lives (Jansen et al., 2013; Namkung et al., 2019). Jain and Dowson (2009), for example, underlined the fact that mathematical comprehension is crucial for personal and professional success. Furthermore, Lipnevich et al. (2016) noted that success in mathematics is related to well-being, satisfaction with life, health, income, employability, and longevity.

Extant research has analyzed the influence of cognitive variables on mathematics achievement, but researchers have paid little attention to the role of emotional or motivational variables (see Miñano and Castejón, 2011). Specifically, these authors found that intelligence did not explain a higher proportion of academic achievement than that provided by variables of an emotional or motivational nature. More recently, García et al. (2016a) concluded that motivation and enjoyment of mathematics were powerful predictors of mathematics achievement. Similarly, Lipnevich et al. (2016) stated that although intelligence was a significant predictor of mathematics achievement, attitudes toward this subject were key to explanation students’ higher achievement. In short, and consistent with Zimmerman (2008), findings indicated that students’ skills and abilities did not offer complete explanations about the magnitude or nature of mathematics achievement. In sum, perceived competence, perceived utility, motivation, and academic achievement may be considered related constructs. For this reason, the present work is aimed to examine the prediction strength of cognitive, motivational, and emotional variables in mathematics achievement, considering students’ gender and age.

### Perceived Competence, Perceived Utility, Motivation, and Emotions

Perceived competence in mathematics is defined as student perceptions about themselves as learners and of their capacity to successfully tackle mathematics tasks. This perception may fit reality to a greater or lesser extent, but in any case, it is a relevant source of students’ motivation (García et al., 2016b). Literature reports a close association between students perceiving themselves as more capable in a particular subject and them being more willing to commit themselves to tasks related to that subject (for example, Pajares, 2008; Cabanach et al., 2009; Rosário et al., 2009). For example, Peixoto et al. (2017) have reported perceived competence to be strongly, significantly, and positively related to mathematics achievement. Similar results have been found with Portuguese students from fifth to ninth grade (Rosário et al., 2012), with British adolescents (Tosto et al., 2016) and with ninth and tenth-grade students from the US (Stevens et al., 2004).

However, feeling oneself to be capable may not be sufficient to explain personal commitment with academic tasks. Furthermore, task commitment needs to be perceived as useful. Perceived utility of mathematics refers to students’ understanding about the applicability and benefits of learning that subject to their lives (Adelson and McCoach, 2011). Findings on the association between the perception of the value of the subject and their ability to learn new concepts and achieve higher in mathematics are mixed, while some researchers found positive relationships (Guy et al., 2005; Jordan et al., 2010). Other studies, did not find a positive association between perceived utility and the use of self-regulated learning strategies to improve the quality of learning (e.g., Cerezo et al., 2019). According to these authors, this could be because students often do not perceive a meaningful relationship between the use of cognitive strategies, high-quality learning, and their academic achievement.

As already noted, students’ involvement in deep learning need both actual and perceived cognitive abilities, but also a strong motivation on the task (Suarez-Alvarez et al., 2014). Motivation for learning may be defined as the initiation of a learning process, the direction set, and the perseverance in path chosen. The relationship between academic motivation and mathematics achievement has been well studied both in elementary (e.g., Mercader et al., 2017) and in high school (e.g., Moenikia and Zahed-Babelan, 2010). Recently, Hammoudi (2019) reported that students more motivated were more willing to find learning opportunities and achieved higher in mathematics than their counterparts. However, and regardless of the theoretical model considered, it is essential to distinguish between motivation for success, or the tendency to succeed in the realization of a task – achievement goals (Wigfield et al., 2015), and intrinsic motivation, or the will to improve mastery on the task – mastery goals (Rodríguez et al., 2001; Murayama et al., 2013). In fact, although both types of motivation are positively related to perceived competence for mathematics (Hammoudi, 2019), intrinsic motivation is related to higher enjoyment of mathematics, higher effort displaced (García et al., 2016a), and higher success rates, even when the difficulty level is high. Recent literature defended the idea that students can pursue both intrinsic and achievement goals. The focus chosen is related to their personal characteristics, the nature of the task, and contextual variables (Wormington and Linnenbrink-Garcia, 2017). In addition, more than 30 years ago, Weiner (1986) found that the strength of students’ motivation to learn was closely related to their reactions to academic successes and failures. Causal attributions may be defined as the explanations people ascribe to their successes and failures and play a determinant effect on students’ motivation and academic achievement (González-Castro et al., 2014). In general, it has been shown that the more adaptive attributional patterns, the more the school achievement (i.e., success is attributed to internal and stable causes, and failure is attributed to changeable, but also internal causes) (Miñano and Castejón, 2011; Miranda et al., 2012).

Finally, learning in general and mathematics tasks in particular are experienced with certain amount of anxiety and a variety of emotional reactions (Rosário et al., 2008). Recently, Chang and Beilock (2016) related motivation with anxiety about mathematics. Math anxiety is the sensation of unease and worry felt when thinking about mathematics or while doing a mathematics task (Buckley et al., 2016). More specifically, math anxiety is characterized by negative feelings toward mathematics, which is likely to result in avoiding mathematics classes and show low math skills (Pizzie and Kraemer, 2017). In summary, many authors have emphasized the strong relationships between math anxiety, motivation, and mathematics achievement, noting that the lower the student’s perceived competence in mathematics, the lower the motivation and the performance in mathematics (Lee and Stankov, 2013; Chang and Beilock, 2016; Passolunghi et al., 2016; Henschel and Roick, 2017).

### Gender and Age

The relationship between cognitive, motivational, and affective variables and achievement in mathematics is significantly influenced by students’ gender and age. Achievement in mathematics seems to vary depending on students’ gender. However, although some researchers have indicated that for 30 years the gender gap in mathematics achievement has been in favor of boys (García et al., 2007; Williams et al., 2016), others have reported that, mainly in countries with equal education for both sexes, boys and girls exhibit few or no differences in mathematical achievement (e.g., Spelke, 2005). As far as our knowledge, there are no data about gender differences regarding the predictive power of cognitive, motivational, or affective variables in relation to mathematics achievement.

When it comes to age, both transversal (e.g., Roskam and Nils, 2007) and longitudinal studies (e.g., Pintrich, 2000; Peetsma et al., 2005), have systematically reported that mathematics results diminish throughout schooling during adolescence. In addition, various studies have indicated that the motivational and emotional variables related to mathematics tend to change over time (Dowker et al., 2016), with perceived competence, perceived utility, intrinsic motivation, and even anxiety diminishing as students go through their schooling (Dowker, 2005; Mata et al., 2012). Nonetheless, as occurs with the gender variable, there is still little information about the interaction between student age and the predictive power of cognitive, motivational, or affective variables in mathematics achievement (Namkung et al., 2019).

### The Current Study

Prior research has been analyzing the relationships between perceived competence, perceived utility, and math anxiety together with motivational variables and academic achievement (Miñano and Castejón, 2011; Lambic and Lipkovski, 2012; Chang and Beilock, 2016). As already noted, literature has reported recurrently positive relationships between mathematics achievement and cognitive competence, perceived competence, motivation (both intrinsic and for success), and adaptive attributional patterns. In addition, negative relationships with anxiety have been reported. However, there is little information about the predictive power of these variables in mathematics achievement when analyzed together. Furthermore, literature lacks information on the effects of the interaction of gender and age while estimating the effect size for each of these variables in mathematics achievement.

Consequently, in this study, we analyze the prediction strength of cognitive, motivational, and affective variables in mathematics achievement, considering students’ gender and age. Grounded on data from previous research, the following hypotheses were set:

1. Cognitive variables (intellectual abilities), along with motivational variables (perceived competence, perceived utility, intrinsic and success motivation, and causal attributions for success and failure), and emotional variables (math anxiety) are good predictors of mathematics achievement.
2. The strength of the association between intellectual abilities and mathematics achievement is lower than that of the motivational or emotional variables.
3. The predictive power of the cognitive, motivational, and emotional variables in mathematics achievement varies depending on the students’ age and gender.

## Materials and Methods

### Participants

Participants in this study were 2,365 secondary school students from various schools in Asturias in the North of Spain. The total high school population (9th to 12th grade) in Asturias is ∼30,000. Data from the international PISA (2018) indicate that adolescents in Asturias scored slightly higher in mathematics than the Organization for Economic Cooperation and Development (OECD) average (Asturias = 491; OECD = 489), but slightly lower than the European Union average (494). The PISA report also indicates that despite boys scored higher in mathematics achievement than girls, the differences were not statistically significant. It is estimated that in the Asturian population as a whole, 7% are immigrants. Data indicate that immigrant students score far below non-immigrants (about 15 points, which is equivalent to a school grade level gap). Concerning socioeconomic status, the PISA report presents Asturias on the OECD average level. There is no evidence of differences between the schools in Asturias (which may be interpreted as an index of educational equality).

The current sample selection procedure was not random; although schools were initially chosen at random, not all agreed to participate. In addition, within the schools, a small number of students declined to participate for various reasons (e.g., being absent in one or more of the evaluation sessions, lack of permission from the family).

The sample subgroups by gender were similar sizes [girls: *n* = 1180 (49.9%); boys: *n* = 1185 (50.1%); *z* = −0.145, *p* = 0.884], although there were significant differences with respect to school grade level [1st year: *n* = 465 (19.7%), 2nd year: *n* = 487 (20.6%), 3rd year: *n* = 731 (30.9%), 4th year: *n* = 682 (28.8%); χ^2^(3) = 92.462, *p* < 0.001]. The gender distribution in each school grade was balanced, with no statistically significant differences: 1st year (50.5% girls; *z* = 0.327, *p* = 0.743), 2nd year (47.8% girls; *z* = −1.345, *p* = 0.178), 3rd year (48.4% girls; *z* = −1.203, *p* = 0.229), and 4th year (52.5% girls; *z* = 1.843, *p* = 0.065). The study did not include students with special educational needs or learning difficulties.

### Instruments

#### Intellectual Abilities

To evaluate students’ intellectual abilities, we used the Triarchic Intelligence Test (STAT). This is a test to measure intellectual abilities according to the Triarchic theory of intelligence (Sternberg, 1993). Its structure is the result of combining the three types of thinking (analytical, creative, and practical) with the content (verbal, numerical, and figurative). Although it is possible to get a score for each subscale, in this study, we only used the total test score. The test has adequate validity and reliability (Sternberg et al., 2001).

#### Motivational and Affective Variables

We measured perceived competence, perceived utility, intrinsic and success motivation, causal attributions, and anxiety from the responses of the students to the Spanish adaptation of the Inventory of Attitudes Toward Mathematics from Fennema and Sherman (1978). In this adaptation, the dimensions used show satisfactory reliability (Cueli et al., 2014): perceived competence (six items, e.g., I believe I can do even the most difficult mathematics tasks; α = 0.85), perceived utility (eight items, e.g., Mathematics is a valuable and necessary subject; α = 0.85), intrinsic motivation (eight items, e.g., I find mathematics enjoyable and stimulating; α = 0.77), motivation for success (five items, e.g., I would like to be one of the best at mathematics; α = 0.86), math anxiety (six items, e.g., Normally, mathematics makes me nervous and uneasy; α = 0.78), attribution of success to internal causes (two items, e.g., I am convinced that to get good grades in mathematics you have to be intelligent; α = 0.71), and attribution of success to external causes (four items, e.g., To get good grades in mathematics, above all you have to be lucky; α = 0.78).

#### Mathematics Achievement

Data about the students’ achievement in mathematics were gathered from the final grades in the subject. The secretaries of the participating schools with the permission of the parents provided data.

### Procedure

The study was conducted in accordance with The Code of Ethics of the World Medical Association (Declaration of Helsinki), which reflects the ethical principles for research involving humans (Williams et al., 2016). The study had the approval of the pertinent Ethical Committee of the Principality of Asturias (reference: CPMP/ICH/135/95, code: TDAH-Oviedo), and all procedures were performed in compliance with relevant laws and institutional guidelines. Data related to the predictor variables (cognitive, motivational, and affective) were collected 3 months before the mathematics tests were taken. Three qualified educational psychologists of the research project visited the schools and collected the data. Parents were informed about the study by the school authorities, and once they were assured of data confidentiality policy, they were asked to sign the informed consent document.

### Data Analysis

Data were analyzed in two stages. Firstly, the descriptive data, correlation matrix, and distribution of means were examined, along with missing values (0.2%). Secondly, we performed various hierarchical regression analyses using SPSS 24. The strength of the associations and effect sizes were evaluated using *R*^2^ (where *R*^2^ *<*0.01 = null; *R*^2^>0.01 and <0.09 = low/slight; *R*^2^>0.09 and <0.25 = medium/moderate; *R*^2^>0.25 = high/strong) and Cohen’s *d* (1988), where *d* < 0.20 indicates a minimal effect size, 0.20 < *d* < 0.50 indicates a small effect size, 0.50 < *d* < 0.80 indicates a moderate effect size, and *d* > 0.80 indicates a large effect size.

## Results

### Preliminary Analysis

Table 1 presents the descriptive statistics and the Pearson correlation matrix. The result of the KMO test indicated that the sampling was adequate (KMO = 0.733), and the Bartlett Sphericity test suggested that the matrix was suitable for multivariate analysis (χ^2^ = 16556.93, *p* <0.001). According to the values for asymmetry and kurtosis, and according to commonly accepted criteria, the variables in the study complied with the criteria for univariate normality (Gravetter and Wallnau, 2014).

**TABLE 1:** Descriptive statistics and Pearson correlation.

	1	2	3	4	5	6	7	8	9	10	11
1. Mathematics achievement	−	0.253**	−0.101**	−0.169**	0.386**	0.339**	0.340**	0.171**	−0.225**	−0.071**	−0.218**
2. Intellectual abilities		−	0.043*	0.226**	0.108**	0.111**	0.073**	0.068**	−0.108**	0.005	−0.135**
3. Gender (0 = girls; 1 = boys)			−	–0.017	0.088**	–0.033	0.087**	–0.007	−0.142**	0.144**	0.165**
4. School year				−	−0.250**	−0.215**	−0.197**	−0.157**	0.146**	0.004	0.071**
5. Perceived competence					−	0.450**	0.517**	0.312**	−0.476**	−0.054**	−0.176**
6. Perceived utility of mathematics						−	0.467**	0.388**	−0.268**	−0.149**	−0.478**
7. Intrinsic motivation							−	0.305**	−0.378**	−0.041*	−0.188**
8. Motivation for success								−	−0.074**	0.110**	−0.177**
9. Math anxiety									−	−0.051*	−0.203**
10. Attribution to internal causes										−	0.318**
11. Attribution to external causes											−
*M*	2.464	11.377	0.50	2.69	3.496	3.582	3.071	3.639	2.843	2.845	2.326
*SD*	1.293	5.471	0.500	1.087	0.852	0.857	0.748	0.980	0.885	1.095	1.095
Asymmetry	0.514	–0.029	–0.004	–0.276	–0.355	–0.078	0.038	–0.437	0.040	0.014	0.511
Kurtosis	–0.875	0.028	–2.002	–1.214	–0.290	–0.514	0.203	–0.327	–0.201	–0.682	–0.686

*p < 0.05; **p < 0.01.

### Prediction of Mathematical Achievement: Overall Sample

The hierarchical regression analysis was performed in three phases: (a) firstly, only intellectual ability was included as the sole predictor (model 1); (b) secondly, gender and school grade level were added as predictors (model 2); and (c) thirdly, perceived competence, perceived utility, intrinsic motivation, motivation for success, attribution of success and failure to internal or external causes, and math anxiety were added (model 3). The results are shown in Table 2.

**TABLE 2:** Results of the regression analysis for the overall simple (*N* = 2365).

	Model 1	Model 2	Model 3
Intellectual ability	0.251***	0.312***	236***
Gender (0 girl, 1 boy)		−0.118***	−0.135***
School year (1st to 4th)		−0.243***	−0.119***
Perceived competence			0.215***
Perceived utility			0.099***
Intrinsic motivation			0.153***
Motivation for success			–0.025
Anxiety			0.010
Internal causal attribution			–0.005
External causal attribution			−0.042*
*R*^2^	0.063	0.131	0.269
Δ*R*^2^		0.068	0.138

*p < 0.05, ***p < 0.001.

Data show that intellectual abilities were strong, positive predictors of mathematics achievement (students with higher intellectual abilities tended to achieve higher results than students with lower intellectual abilities). Nonetheless, although the amount of explained variance was low (6.3%), the predictive capacity was similar in the two subsequent models, with a moderate effect size (*d* = 0.538). In fact, the predictive capability hardly suffered as a consequence of the inclusion of gender and school grade level (model 2) or the motivational and affective variables (model 3).

Gender and school grade level were also predictors of mathematics achievement, with a low percentage of explained variance (and both with small effect sizes: *d* = 0.307 and *d* = 0.257, respectively). This association was stable even after including the motivational and affective variables in the regression model (model 2 vs. model 3). Girls tended to show higher mathematics achievement than boys, *F*_(1,2365)_ = 24.234; *p* < 0.001; η^2^ = 0.010, although the effect size for these differences was small (*d* = 0.20). As students progressed through the school years, their mathematics achievement tended to fall, *F*_(3,2381)_ = 30.261; *p* <0.001; η^2^ = 0.037, again with a small effect size (*d* = 0.39).

Data indicated that including the motivational and affective variables in the model was statistically significant, *F*_(2, 2354)_ = 63.341; *p* <0.001, with a moderate strength for the association: *R*^2^ = 0.14. From the seven variables included in the third model, the only predictors of mathematics achievement were perceived competence (albeit with a small effect size; *d* = 0.39), the perceived utility of mathematics (with a very small effect size; *d* = 0.17), intrinsic motivation (again with a small effect size; *d* = 0.29), and the attributions of successes and failures to external causes for which, although statistically significant at *p* < 0.05, the size of the coefficient of prediction was not significant (*d* = 0.08). Neither motivation for success, nor attribution to internal causes, nor anxiety were found to be predictors of mathematics achievement.

Finally, model 3 predicted a significant and relevant amount of the variability of mathematics achievement (with a large effect size: *R*^2^ = 0.27). Nonetheless, it is important to note, as the data in Table 2 shows, that while intellectual abilities explained a small amount of the variance in mathematics achievement (*R*^2^ = 0.063), the motivational and affective variables explained a moderate amount of the variability in achievement (*R*^2^ = 0.138).

### Prediction of Mathematics Achievement by Gender

Table 3 presents the results of the regression analysis for the girls’ and boys’ samples. Data were similar for both subsamples and did not differ significantly from what has already been reported for the overall sample. Specifically, we learned that intellectual abilities, despite the low amount of variance explained (*R*^2^ = 0.050 girls; 0.080 boys), were good predictors of mathematics achievement in both samples, even after the inclusion of the other variables. Likewise, the perceived capability for mathematics explained academic achievement to the same extent for boys and girls, with similar results for intrinsic motivation. However, perceived utility has not significantly predicted mathematics achievement for the girls sample, which was not true for boys. Irrespective of the samples, for the other variables (i.e., motivation for success, attributional processes, and mathematics anxiety), data were not found to be significantly associated with mathematics achievement.

**TABLE 3:** Results of hierarchical regression models for the variable gender.

	Model 1	Model 2	Model 3
**Girls (*n* = 1180)**			
Intellectual ability	0.230***	0.280***	0.204***
School year (1st to 4th)		−0.232***	−0.110***
Perceived competence			0.203***
Perceived utility			0.067
Intrinsic motivation			–0.008
Motivation for success			0.136***
Anxiety			0.051
Internal causal attribution			–0.034
External causal attribution			–.022
*R*^2^	0.053	0.104	0.228
Δ*R*^2^		0.051	0.124
**Boys (*n* = 1185)**			
Intellectual ability	0.283***	0.346***	0.270***
School year (1st to 4th)		−0.257***	−0.129***
Perceived competence			0.239***
Perceived utility			0.136***
Intrinsic motivation			–0.047
Motivation for success			0.167***
Anxiety			–0.036
Internal causal attribution			–0.048
External causal attribution			–0.035
*R*^2^	0.080	0.143	0.306
Δ*R*^2^		0.062	0.164

***p < 0.001.

Finally, analyzing the coefficients of determination, we found that the three models explained a higher quantity of the variance in the boys than in the girls sample, with the effect size being moderate for the girls (*R*^2^ = 0.228) and large for the boys (*R*^2^ = 0.306) sample. In both samples, the amount of variance explained by intellectual abilities was small, while the variance explained by the motivational and affective variables was moderate (*R*^2^ = 0.124 girls; 0.164 boys).

### Prediction of Mathematical Achievement by School Year

Table 4 presents the results of the predictions of mathematics achievement in the four school grade levels. The following are some of the most interesting results.

**TABLE 4:** Results of hierarchical regression models for the variable school year.

	Model 1	Model 2	Model 3
**School year: 1st year (*n* = 465)**			
Intellectual ability	0.403***	0.408***	0.305***
Gender (0 girl, 1 boy)		−0.085***	–0.058
Perceived competence			0.222***
Perceived utility			0.089
Intrinsic motivation			–0.029
Motivation for success			0.068
Anxiety			0.019
Internal causal attribution			−0.162***
External causal attribution			–0.031
*R*^2^	0.162	0.169	0.344
Δ*R*^2^		0.070	0.175
**School year: 2nd year (*n* = 487)**			
Intellectual ability	0.367***	0.372***	0.260***
Gender (0 girl, 1 boy)		−0.143***	−0.183***
Perceived competence			0.149***
Perceived utility			0.078
Intrinsic motivation			–0.013
Motivation for success			0.162***
Anxiety			0.130***
Internal causal attribution			−0.119***
External causal attribution			0.075
*R*^2^	0.134	0.155	0.323
Δ*R*^2^		0.021	0.169
**School year: 3rd year (*N* = 731)**			
Intellectual ability	0.259***	0.257***	0.204***
Gender (0 girl, 1 boy)		−0.126***	−0.154***
Perceived competence			0.271***
Perceived utility			0.106***
Intrinsic motivation			–0.013
Motivation for success			0.107***
Anxiety			–0.010
Internal causal attribution			0.052
External causal attribution			–0.024
*R*^2^	0.067	0.083	0.223
Δ*R*^2^		0.016	0.140
**School year: 4th year (*n* = 682)**			
Intellectual ability	0.250***	0.264***	0.207***
Gender (0 girl, 1 boy)		−0.117***	−0.139***
Perceived competence			0.184***
Perceived utility			0.073
Intrinsic motivation			–0.039
Motivation for success			0.213***
Anxiety			–0.056
Internal causal attribution			–0.041
External causal attribution			0.036
*R*^2^	0.062	0.076	0.206
Δ*R*^2^		0.014	0.130

***p < 0.001.

Firstly, as students’ progress through the school years, up to the third year, there was a significant fall in the importance of intellectual abilities while explaining mathematics achievement. In first and second years, the amount of variance was moderate (*R*^2^ = 0.162 in 1st year; 0.134 in 2nd year), but small in the third and fourth years (*R*^2^ = 0.067 in 3rd year; 0.062 in 4th year). At the same time, perceived competence was a significant predictor of mathematics achievement in all four school years, and there was no decrease over time. Secondly, we found that intrinsic motivation was also a good predictor of achievement, except in the first year, in which this relationship was not statistically significant. The remaining motivational and affective variables were not clear, consistent predictors of mathematics achievement in the four school years. Taken together, and considered as a trend, the variance explained by motivational and affective variables decreased as students progressed from the 1st to 4th year high school grades. For the four school grade levels, the size of the association between the predictor variables and mathematics achievement was moderate or medium (17.5, 16.9, 14, and 13% of the variance explained, respectively). Thirdly, we also found that, in general, the explained variance for mathematics achievement was higher for the first two school years (34.4 and 32.3% of the variance explained for mathematics achievement) than for the last two (22.3 and 20.6% of variance explained, respectively). For the first two school grade levels, the size of the association was large and moderate for the final two. Finally, regarding gender, with the exception of the first year, in which the association was not statistically significant, for the other three school grade levels, girls tended to be more likely in showing higher mathematics achievement than boys (although the effect size was small in all cases).

## Discussion

In this study, we aimed to assess the predictive capacity of a set of variables: cognitive variables (intellectual ability), motivational variables (perceived competence, perceived utility, intrinsic motivation, motivation for success, and attribution of causality for success and failure), and emotional variables (math anxiety) in determining students achievement in mathematics. Our goal was focused on determining not only their explanatory power but also to further understand their interactions with the variables gender and school grade level. Although vast research has examined the predictive capacity of one or more of these variables, there are not much data analyzing them together, nor addressing whether the resulting predictive models would vary depending on variables such as gender and school grade level.

We formulated various hypotheses based on previous research. Firstly, we hypothesized that cognitive variables, motivational variables, and affective variables are good predictors of mathematics achievement. We also hypothesized that the size of the association between intellectual abilities and mathematics achievement is smaller than the size of the association between the motivational and emotional variables. Current data partially supported this general hypothesis.

In general terms, we found that students tended to be more likely to perform well in mathematics tasks when they had better intellectual abilities, higher perceived competence for mathematics, higher intrinsic motivation (i.e., interest in understanding mathematics and becoming more expert), and when they perceived mathematics to be useful. In line with previous research (e.g., Stevens et al., 2004; Miñano and Castejón, 2011; Lambic and Lipkovski, 2012; Miñano et al., 2012; Rosário et al., 2012; Tosto et al., 2016; Hammoudi, 2019), data showed the relationship of intellectual abilities and motivational variables (particularly perceived competence and intrinsic motivation, and perceived utility to a lesser extent). In addition, similarly to other studies (e.g., Miñano and Castejón, 2011; Miñano et al., 2012; García et al., 2016b; Lipnevich et al., 2016; Gilar-Corbi et al., 2019), we also concluded that the motivational variables were stronger predictors of mathematics achievement than the students’ intellectual abilities.

In this regard, there are some aspects worth noting. Firstly, the fact that when it comes to explain student’s achievement, their perceived capabilities are as important as their actual abilities (see also, Erturan and Jansen, 2015). This is interesting because perceived competence is a personal construction, and therefore can be modified according to student’s experiences with mathematics. For this reason, teachers could consider helping students on their work, which offers the chance of successfully constructing confidence to tackle challenges and improve learning in mathematics. Secondly, it seems that at these ages, students still trust that what they learn in mathematics class will be useful; on the contrary, findings from Cerezo et al. (2019) indicate that college students fail to see the utility of what they are learning as a significant variable to organize their learning behaviors. For this reason, teachers and school administrators may wish to consider teaching learning strategies to help students link what they are learning with the near future (Cabanach et al., 2009; Rosário et al., 2015). Thirdly, as expected (e.g., Miñano and Castejón, 2011; García et al., 2016a; Lipnevich et al., 2016), the interest in learning a subject, such as mathematics, was associated with positive results. However, this did not happen, as our data showed, when learning mathematics was understood as an opportunity to outshine others or to gain some kind of reward. For this reason, the design of appropriate instructional strategies should include not only tasks focused on increasing students’ self-confidence, and likely to be perceived as useful, but also tasks likely to increase students’ interest and encourage them to deep their learning and compete with themselves rather than with their peers (Rosário et al., 2013).

Nonetheless, in contrast to some previous studies, the variables of a more emotional nature were not shown to be predictors of mathematics achievement (in either boys or girls samples), except in the second year of high school, in which anxiety and causal attribution processes significantly predicted mathematics achievement, thought. There may be various explanations for this.

When it comes to math anxiety, as mentioned in the beginning of this paper, prior data suggested a significant, strong, and negative relationship between anxiety and mathematics achievement (e.g., Rosário et al., 2008; Ashcraft and Moore, 2009; Miñano and Castejón, 2011; Maloney and Beilock, 2012; Miranda et al., 2012; Suárez-Pellicioni et al., 2015; Chang and Beilock, 2016; Passolunghi et al., 2016; Henschel and Roick, 2017). Firstly, and despite data from our study are not consistent with those results, they are in line with findings from Erturan and Jansen (2015), showing that when data are analyzed with regression equations, which include studying anxiety together with other variables (e.g., perceived competence for mathematics as predictors of mathematics achievement), anxiety is no longer predictive of mathematics achievement. Secondly, the magnitude of the relationship between anxiety and mathematics achievement could be affected by which dimension of anxiety is examined (Mammarella et al., 2018). Specifically, Dowker et al. (2016), and Henschel and Roick (2017) noted that the cognitive and affective dimensions of anxiety could be differently related to mathematics achievement. Similarly, Goetz et al. (2013) and Bieg et al. (2015) observed high levels of trait anxiety about mathematics, girls scoring higher, but this did not happen with state anxiety. Our findings could be related to the fact that the items of the questionnaire used, although not referencing very specific situations, could be understood as more related to state anxiety than to trait anxiety. Thirdly, another possible explanation may be related to the role of anxiety in the association with mathematics achievement and other variables such as perceived competence for mathematics (Erturan and Jansen, 2015). In a recent study, Pérez-Fuentes et al. (2020) attempted to learn whether mathematics anxiety, rather than directly predicting mathematics achievement, functioned as a mediating or moderating variable for other variables involved. In that study, they attempted to learn whether the relationship between perceived math ability and math achievement was mediated, at least partially, by anxiety, and whether it may even differ (in intensity or direction) depending on anxiety levels. Their results indicated that anxiety partially mediated, and moderated, the relationship between perceived competence and achievement. In terms of the moderating role, Pérez-Fuentes et al. (2020) found that when mathematics anxiety was high, the effect size of perceived competence for mathematics was large, whereas with low levels of anxiety, the effect was small. Authors suggested that when students experience high levels of math anxiety, the importance of their confidence in themselves grows significantly as a determinant of mathematics achievement. In contrast, when anxiety is low, students’ self-confidence is a much less strong determinant of achievement. However, more research is needed to confirm these findings.

The third hypothesis raised the possibility of the influence of gender and age on the predictors of mathematics achievement and of the magnitudes of these relationships. The direction of the impacts could not be further specified due to the limited available knowledge. In fact, to the best of our knowledge, available data only relates gender differences to some of the variables analyzed in the current study. For example, mathematics achievement (Spelke, 2005; García et al., 2007; Reilly et al., 2015; Fahle, 2016; Williams et al., 2016), achievement depending on the school grade level (Fahle, 2016), mathematics anxiety (Hill et al., 2016; Henschel and Roick, 2017) and perceived competence for mathematics (Henschel and Roick, 2017).

Regarding gender, data from our study add literature as follows. Firstly, there were no relevant gender differences regarding the predictor variables for mathematical achievement (i.e., intellectual abilities, perceived competence, or intrinsic motivation), although for boys, unlike the girls, the perceived utility of mathematics has shown to be a significant and positive predictor of mathematics achievement. Secondly, the variability in mathematics achievement that could be explained by the predictors was substantially higher in boys (large effect size) than in the girls (moderate effect size) sample. Thirdly, we found that in both samples, the predictive capacity of the non-cognitive variables (mainly perceived competence for mathematics and intrinsic motivation) was substantially higher than that shown by cognitive abilities (intellectual abilities). Whereas the non-cognitive variables exhibited a moderate predictive capacity, a small association was found for the cognitive abilities. Finally, it is worth noting that, consistent with recent studies (e.g., Erturan and Jansen, 2015), we did not find gender differences related to the magnitude of the association between anxiety and mathematics achievement, although there were differences in the direction of the relationship (positive for boys, negative for girls). As in the study by Erturan and Jansen (2015), in our research, perceived competence strongly and positively predicts performance in mathematics, for both boys and girls, but anxiety does not. So, we can conclude with Erturam and Jansen that “perceived math competence is more important in predicting performance than math anxiety” (p. 431).

With respect to the school grade level, this study adds literature by showing a decrease in the level of some of the variables taken as students’ progress (e.g., a decrease in perceived competence, motivation, perceived utility of mathematics, and mathematics achievement; Peetsma et al., 2005; Roskam and Nils, 2007; Mata et al., 2012; Regueiro et al., 2015; Dowker et al., 2016). To be specific, we found that as students’ progress throughout high school, the cognitive, motivational, and affective variables taken explain less of mathematics achievement. These findings indicate that mathematics achievement progressively depends less on the personal variables examined (e.g., intellectual abilities, perceived competence, motivation, anxiety, and attributional processes) and more on other variables: personal (e.g., personal engagement) and non-personal (e.g., school and family variables). In fact, despite the fact that it is reasonable to think that students’ learning and achievement depend to a certain extent on family, school, and teaching variables, it is also expected that the main strong factors would be those personal to the students themselves (cognitive, motivational, and emotional). Thus, although the cognitive, motivational, and emotional variables considered in this study explain a significant proportion of the variability in mathematics achievement (with a large effect size), 70% of the variance remains to be explained. Although it may seem like a key strength of this study, it is clearly a shortcoming, since the remaining 70% have cleared educational and research implications. It does not seem feasible that 70% of adolescents’ mathematics achievement can be explained by variables external to the student. It is reasonable to think that the different non-personal conditions (family, school, and teaching) may be important in students’ learning and development, but through their influence on student variables (e.g., mainly those that can be changed, such as perceived competence, motivation, attitudes toward mathematics, attributional processes, anxiety) rather than separately from them. Future research, perhaps through causal relationship models, preferably with longitudinal, or repeated measure designs, should examine this idea more deeply.

In sum, considering the results of the present work, there are some educational implications that is necessary to highlight. First, if teachers focus in the cognitive skills of students in order to analyze or predict their academic results, they would be omitting important factors as their motivational situation. In this sense, beyond other variables of emotional nature, working on the perceived competence, intrinsic motivation, and perceived utility of students could have a positive impact on their mathematics achievement, especially in the first years of high school. Also, teachers have to consider other variables in their professional practice (e.g., family environment, instructional processes or math, or practice implicit theories).

Finally, it is important to note that despite the fact data in this study collected data from a wide sample of students and were representative in terms of gender and school grade level, it should be taken with caution when generalizing to different educational communities or to societies with substantially different educational systems. Nonetheless, the fact that mathematics is important for all of the OECD countries might reduce the likelihood of bias in generalizing these results. It is also essential to bear in mind that data about motivational and affective variables were collected by self-reports, which may bring bias. However, most of the research reviewed also used self-reported data, which should facilitate comparison.

## Data Availability Statement

The datasets generated for this study are available on request to the corresponding author.

## Ethics Statement

The studies involving human participants were reviewed and approved by Committee of the Principality of Asturias. Written informed consent to participate in this study was provided by the participants’ legal guardian/next of kin.

## Author Contributions

JN, AA, and CR contributed conception and design of the study. MC and TG organized the database. JN and PR performed the statistical analysis. CR, AA, and MC wrote the first draft of the manuscript. PR, TG, and JN wrote sections of the manuscript. All authors contributed to manuscript revision, read and approved the submitted version.

## Conflict of Interest

The authors declare that the research was conducted in the absence of any commercial or financial relationships that could be construed as a potential conflict of interest. The reviewer MC declared a past co-authorship with one of the authors PR to the handling Editor.
